# Supplementary material for: Causal interplay between lactose intolerance and gut microbiota: a combined bidirectional Mendelian randomization and in vivo validation study
Source: Front Nutr. 2026 Jun 1;13:1803337. doi: 10.3389/fnut.2026.1803337 (PMC13265576; doi:10.3389/fnut.2026.1803337)
Supplement: Supplementary file 1 [file Data_Sheet_1.zip › supplementary materials/Forward/leave one out cross validation/ebi-a-GCST90027581.finngen_R12_E4_LACTONAS.pdf]

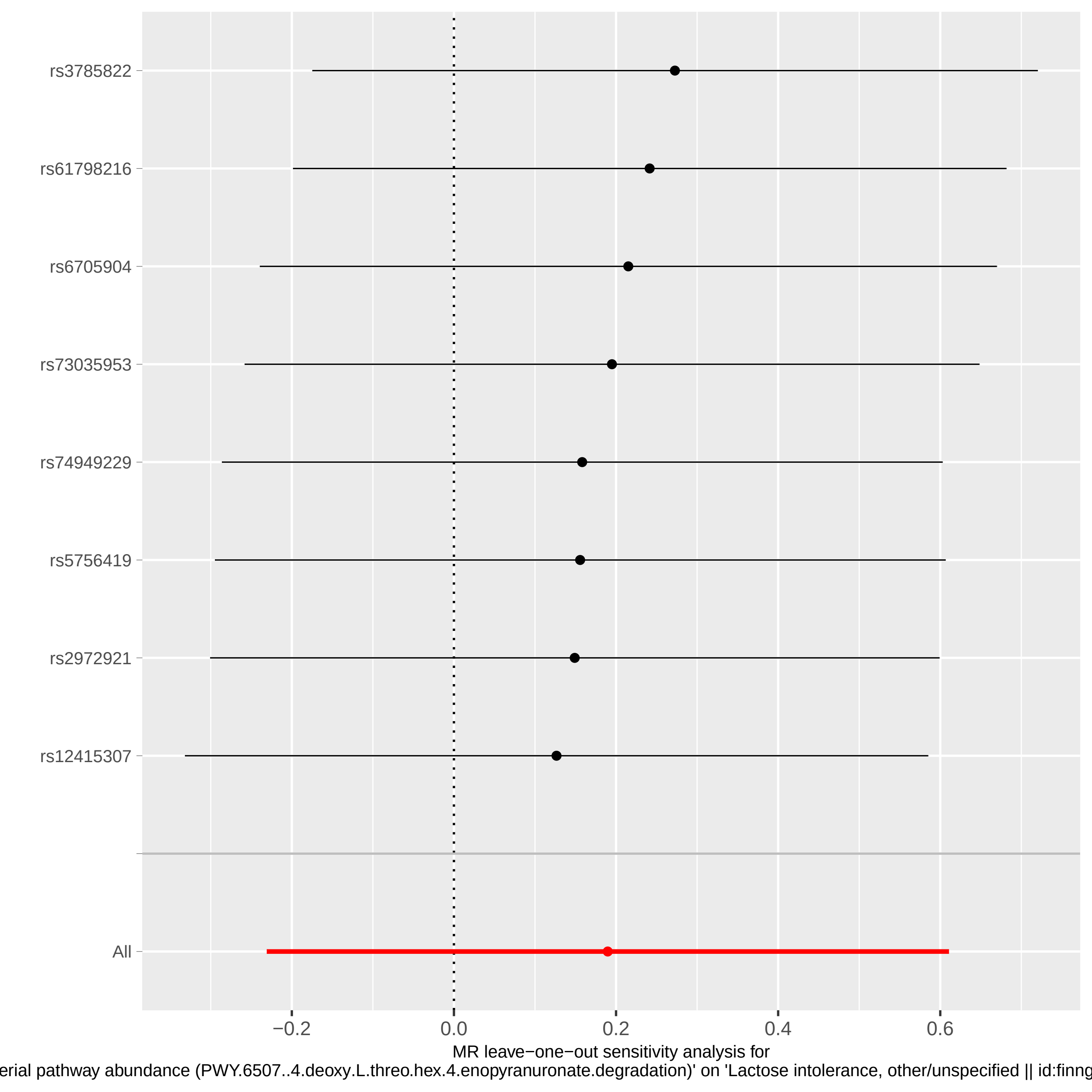
rs3785822
rs61798216
rs6705904
rs73035953
rs74949229
rs5756419
rs2972921
rs12415307
All
−0.2
0.0
0.2
0.4
0.6
MR leave−one−out sensitivity analysis for
erial pathway abundance (PWY.6507..4.deoxy.L.threo.hex.4.enopyranuronate.degradation)' on 'Lactose intolerance, other/unspecified || id:finng
